# Effects of endocrine disruptors on the neurological system

**DOI:** 10.55730/1300-0144.6127

**Published:** 2025-11-11

**Authors:** Kadircan KARATOPRAK, Soner CANDER

**Affiliations:** Division of Endocrinology and Metabolism, Department of Internal Medicine, Faculty of Medicine, Bursa Uludağ University, Bursa, Turkiye

**Keywords:** Endocrine disruptors, neurological diseases, Parkinson’s disease, Alzheimer’s disease, neuroendocrine effects, neurobehavioral and neurodevelopmental effects

## Abstract

**Background/aim:**

There is increasing interest in endocrine disrupting chemicals because of the potential effects on neurological health. These chemicals are widely found in various consumer products and industrial processes, and can lead to serious disorders of the endocrine system by disrupting hormone synthesis, expression, and function. The aim of this review was to examine epidemiological and experimental findings by investigating the link between exposure to endocrine disrupting chemicals and adverse neurological outcomes.

**Materials and methods:**

In the preparation of this review, a PubMed literature search was conducted using the words “endocrine disruptors,” “neuroendocrine effects,” “neurobehavioral effects,” and “neurodevelopmental effects” and articles containing relevant studies were examined.

**Results:**

Recent studies have shown a strong correlation between exposure to endocrine disrupting chemicals and the development of neurodegenerative diseases such as Alzheimer’s and Parkinson’s disease, and neurodevelopmental diseases such as autism spectrum disorder and attention deficit hyperactivity disorder. The effects of common pollutants such as pesticides, bisphenol A, polychlorinated biphenyls, and heavy metals on the endocrine system have been especially emphasized.

**Conclusion:**

In conclusion, understanding the role played by endocrine disrupting chemicals in the development of neurological diseases will be of critical importance in the development of new strategies to prevent these diseases.

## 1. Introduction

The effects of endocrine disruptors on human health have been investigated in recent years. Endocrine disruptors are chemical substances widely found in the environment and generally originate from industrial processes. They are present at low concentrations in many natural and manufactured products such as toys, furniture, medical devices, and cosmetics, and are among the most common pollutants in the environment. They may also be found naturally in some plant-based products. People and animals may be exposed to these chemicals through various pathways. For example, there can be transfer from mother to fetus through food and water consumption; absorption through the skin, respiratory pathway, or placenta; or from mother to infant through breastfeeding [1]. These chemicals are exogenous agents that have negative effects on the endocrine system by disrupting the synthesis, expression, transport, metabolism, and function of hormones. Epidemiological studies have shown that these chemicals are associated with diseases such as obesity, diabetes, cancer, and infertility, and that intrauterine exposure in particular plays an important role in the development of these diseases [2,3]. Neurological diseases emerge as a result of the complex interaction of genetic and environmental factors. As the etiologies have not been fully clarified, the relationship between endocrine disruptor chemicals and many neurological diseases such as neuroendocrine diseases, Alzheimer’s disease (AD), Parkinson’s disease (PD), and neurodevelopmental disorders, has become one of the most researched subjects. Research in this field is of increasingly greater importance to understand the effects of endocrine disruptors on neurological health.

## 2. Association with neurological diseases

Neurological diseases encompass various disorders that affect the central and peripheral nervous systems. By disrupting the functions of the brain, spinal cord, nerves, nerve roots, autonomous nervous system, and neuromuscular attachments and muscles, these diseases lead to losses in motor, sensory, cognitive, and autonomic functions. The commonly seen diseases in this group include AD, PD, multiple sclerosis, epilepsy, migraine and other headache disorders, stroke, neuroinfections, traumatic brain injuries, and various neurological disorders^1^.

In the last quarter century, there has been an increase in the prevalence of neurological diseases in parallel with the production of endocrine disruptors, and this tendency is thought to be connected to increasing environmental pollution [4]. Therefore, the identification of these substances that can lead to neurological diseases and increasing awareness of this information by reaching a large mass, is of critical importance in terms of developing strategies to reduce exposure and prevent these diseases.

Various studies have shown that exposure to endocrine disruptors create negative effects on memory, learning, attention, perception, and neurological development. These chemicals can have agonist and antagonist effects on neurotransmitters, neurohormones, and neuropeptides that regulate the neuroendocrine system. They can even cause epigenetic changes such as DNA methylation and/or acetylation and histone modifications [5]. As these types of chemicals have the potential to disrupt the normal functioning of the neuroendocrine system, they are known as neuroendocrine disruptors. There is a wide range of endocrine disruptors that have an effect on neurological functions and are known to be associated with disorders such as AD, PD, dementia, and hyperactivity disorder. These substances include diethylstilbestrol, organophosphate pesticides, phytoestrogens, phthalates, perchlorate perfluoroalkyl substances (PFAS), benzophenones, parabens, bisphenol A (BPA), trichlosane, dioxins, polychlorinated biphenyls (PCB), polybromide diphenyl ethers, octamethylcyclotetrasiloxane (D4), and heavy metals [6]. Of these chemicals, BPA, which has both estrogenic and antiandrogenic effects, is the most widely encountered endocrine disruptor and a synthetic xenoestrogen. Endocrine disruptors generally have a lipophilic structure and are resistant to metabolic processes.

The effects of endocrine disruptors can vary depending on the type, timing, and duration of exposure. It is difficult to determine where specifically in the body these pollutants target because people are generally exposed to more than one disruptor simultaneously. Moreover, factors such as the chemical structure of environmental hormones, and the age, sex, and other personal characteristics of the individual increase the range of responses given to these effects, which makes the identification of specific effects more complicated. Both animal models and human studies have shown that the mechanisms affected by endocrine disruptors are included in more than one pathway. By mimicking the functions of endogenous hormones, endocrine disruptors can increase or decrease cellular responses. At the same time, they can prevent the effects of hormones or disrupt the hormone balance by stimulating and suppressing hormone production. These processes allow intervention in the synthesis, transport, effects, and destruction of hormones [7]. Endocrine disruptors can show their effects through nuclear receptors, nonsteroid receptors, transcription coactivators, and various enzymatic pathways that regulate cellular functions. Endocrine disrupters can disturb hormone balance, modulating intracellular signaling and gene expression.

Endocrine disruptors can have neurotoxic effects through estrogen, androgen, and thyroid nuclear hormone receptors. Disruptions to neurotransmitter signaling processes can lead to changes in critical biomarkers such as receptors, calcium homeostasis, and oxidative stress. Endocrine disruptors, polychlorinated biphenyls (PCBs) in particular, cause changes in dopamine, serotonin, and acetylcholine levels. These changes can create profound effects on neurological functions by affecting cellular signal pathways such as intracellular calcium buffering, arachidonic acid expression, synaptosome calcium intake, and protein kinase C (PKC) activity [8,9]. Through a continuous phosphorylation process, PKC causes nuclear transcription and consequently changes proteome activity. PKC is a signaling molecule that is important in neuron development and death. It is also a target PCB, causing neurotoxicity [10]. In addition, many endocrine disruptors increase reactive oxygen species (ROS). ROS in the mitochondria in the brain is closely associated with neurodegenerative diseases. Neurons are long living, undifferentiated cells. This means an increase in ROS levels and decrease in antioxidant enzymes can disrupt the oxidation–reduction balance and trigger neuron death. Brain-derived neurotrophic factor (BDNF) plays a critical role in protection against neurological diseases. BDNF not only supports the development and renewal of neurons, but also protects against neurodegenerative diseases by increasing the functionality of neurotransmitters [11]. Exposure to neurotoxic heavy metals (e.g., lead, mercury, arsenic, and cadmium) can negatively affect BDNF synthesis and can lead to a decrease in serum BDNF synthesis [11]. All these processes have been reported to play a role in the development of AD, PD, and Huntington’s diseases.

## 3. Neurological diseases

### 3.1. Parkinson’s disease

PD is the most common cause of Parkinsonism syndrome. It is one of the most common neurodegenerative diseases in adults, characterized by the loss of dopaminergic neurons in the brain, especially in the substantia nigra region. It manifests with symptoms such as tremor at rest, bradykinesia, rigidity, and postural imbalance, and is accepted worldwide as one of the leading causes of neurological morbidity and mortality. Although the cause of neurodegeneration and the etiological trigger of PD are still not known, there are various hypotheses related to alpha synuclein misfolding, aggregation and toxicity, mitochondrial dysfunction, erroneous proteolysis, oxidative stress damage, neuroinflammation, and genetic factors [12].

Heavy metals and trace elements can show both physiological and pathological effects on the nervous system. Excessive exposure to toxic metals such as mercury, zinc, lead, iron, copper, aluminum, manganese, cadmium, selenium, and arsenic can lead to dopaminergic neuron degeneration by crossing the blood–brain barrier [13].

The main sources of metal-related PD are environmental pollution, occupational exposure, polluted seafood, drugs, and metal dental restorations such as amalgam fillings. High levels of heavy metals accumulating in the brain increase the formation of alpha synuclein by triggering oxidative stress and mitochondrial dysfunction, and this leads to dopaminergic neuron damage. There is increasingly more evidence that accumulation of these metals, which are found at very low levels in the normal body, initiates free radical production and disrupts basal ganglion signaling [14]. Long-term heavy metal exposure leads to an increase in inflammatory cytokines such as IL-1β, IL-6, and TNF-α, and can cause neuroinflammation and eventually neuron loss. In individuals with over 20 years of occupational exposure to lead, copper, and manganese, the risk of PD was reported to be 2–10-fold higher [13]. Lead has a long half-life and approximately 90% is accumulated in the bones. Two studies that compared the bone lead levels in PD patients and healthy individuals showed that a one-unit increase in the bone lead level could increase the risk of PD by 2–3-fold [14].

Long-term exposure to low-dose pesticides can affect brain cells. A metaanalysis showed that exposure to herbicides and insecticides increased the risk of PD by 1.6-fold, and this risk increased up to 2.5-fold in individuals with occupational exposure [15]. Methyl-phenyl-tetrahydropyridine (MPTP) is a toxin that kills dopaminergic neurons in the brain, leading to PD-like symptoms. Paraquat (a bipyridilium derivative) is an herbicide frequently used in cotton and sunflower fields. It has structural similarity to MPTP. Therefore, paraquat and similar pesticides are thought to play a role in the development of PD. Exposure to maneb and paraquat together in particular has been reported to increase the risk of PD by 4.2-fold [16]. Dieldrin, which is an organochloride pesticide, shows selective toxic effects on dopaminergic neurons, increase ROS levels in nigral dopaminergic neurons, activate microglia, inhibit mitochondrial phosphorylation, and trigger alpha synuclein protein aggregation [17].

Exposure to BPA has been reported to lead to a decrease in acetylcholine esterase activity and dopamine levels, but there has been no longitudinal clinical study on the BPA exposure of PD patients and the role of this chemical in the etiology of the disease [14].

### 3.2. Alzheimer’s disease

The etiology and pathogenesis of AD are not fully known. It is a neurodegenerative disease especially prevalent in older adults and is the most common cause of dementia. Risk factors include age, genetic predisposition, diabetes, hypertension, dietary habits, smoking, lack of physical exercise, and exposure to environmental toxins. The neuropathological features that differentiate AD are diffuse and neritic plaque characterized by extracellular accumulated amyloid beta (Aβ), and neurofibril balls formed with hyper phosphorylated tau (p-tau) protein accumulation within the cell [18].

Pesticides are known neurotoxins. Although the mechanisms related to the effects of pesticides on neurodegenerative diseases have not been fully established, lengthy or low-dose exposure to pesticides such as paraquat, dieldrin, organochlorine, and organophosphates have long been suspected of playing a role in the development of these diseases. Organophosphate and organochloride pesticides can cause permanent damage to the nervous system by blocking acetylcholinesterase in the somatic, autonomic, and central nervous system synapses [19]. Many studies have shown that exposure to pesticides, and environmental and occupational heavy metals could be an important risk factor for AD. Recent biomarker studies have reported that even at a low-level of pesticide exposure, detectable levels have been reached in the circulation and this increased the risk of disease. In a case–control study of 86 pathologically confirmed AD patients and 79 healthy control subjects, dichlorodiphenyldichloroethylene (DDE) levels in the serum of AD patients were 3.8-fold higher than those of the control group [20]. A metaanalysis conducted in 2019 reported a 50% increased risk of AD, and a 65% increased risk of PD in pesticide users [21].

Metals have been widely examined through biomarkers, especially in case–control studies. However, the strength of the evidence is generally low [22]. Heavy metals such as arsenic, lead, and cadmium increase Aβ production in the hippocampus and cortex, tau phosphorylation, and amyloid protein precursor expression [23]. Of the common pollutants in the general population, the strongest epidemiological evidence to date is for fine particle materials <2.5 μm in diameter (PM 2.5) found in the air [24, 25]. An increase of 10 μg/m^3^ in PM 2.5 air concentration (USA Environment Protection Agency annual standard) can increase the risk of all dementias by approximately 30%, AD by 65%, and PD by 15% [25].

## 4. Neuroendocrine effects

Neuroendocrine systems protect homeostasis of the organism and regulate vital functions such as stress, reproduction, growth, and metabolism through interaction with both the central nervous system and peripheral endocrine systems. As neuroendocrine cells in the brain have both neuronal and endocrine characteristics, this makes the neurotoxic and neurobiological effects of endocrine disruptors more widespread and complex. Therefore, in addition to endocrine problems, exposure to endocrine disruptors can lead to serious health problems such as irregularities in stress response and neurodegenerative diseases. By targeting neuroendocrine centers, the hypothalamus in particular, endocrine disruptors can disrupt hormone signaling and have severe effects on these neurobiological processes. This is because the hypothalamo–hypophysis axis directs the basic hormonal processes in the regulation of thyroid, adrenal glands, gonads, somatic growth, and other vital functions. These disruptors may also affect steroid hormone receptors that are widespread in the brain, and other signaling pathways. Exposure, especially in developmental periods, is of critical importance and even small changes in hormones in this period can have large effects on neurobiological outcomes. However, most research in this area is based on animal studies, because methods such as postmortem analyses of samples or sensitive measurements of hypothalamic hormones are not possible in human studies for practical and ethical reasons.

BPA is found in food packaging, toys, resins used in the lining of many canned foods and drinks, spectacle lenses, sports safety equipment, dental monomers, and medical equipment. Animal studies have shown that exposure to BPA affects mRNA expression and that estrogen receptor alpha and beta (ERα and ERβ) proteins in different areas of the brain are affected in various ways. The effects of BPA on estrogen levels can vary depending on the region [26, 27]. In a study of male and female adult rats treated with low-dose BPA for 4 days, there were significant changes in mRNA expression of 5α-reductase 1 enzymes and aromatase in the prefrontal cortex. The aromatase mRNA levels in both sexes increased, while a decrease was recorded in 5α-reductase 1 mRNA expression in female rats. These findings showed how BPA could change gene expression in brain regions in a sex-specific manor [28]. Exposure to BPA can also increase adrenal weight and weaken the stress response [29].

Phthalates are a large class of artificial, multifunctional chemicals used in the production of many industrial and consumer goods such as toys, plastics, coatings, cosmetics, and medical products. By altering the expression of genes associated with peroxisome proliferator-activated receptors (PPAR), exposure to these chemicals may lead to obesity [30]. By affecting estrogen receptor (ER) signaling, there could be estrogenic effects similar to those of BPA, but this effect is weak and controversial [31]. However, they can cause disrupt thyroid homeostasis, decreasing thyroid hormone levels. The activated Ras/Akt/TRHr pathway and hepatic enzymes play a vital role in the thyroid disrupting effects of phthalates [32].

PCBs are used as insulating liquids in electrical equipment and sealant materials, in adhesives, and as additives in paints and inks. polybrominated diphenyl ethers (PBDEs) are used as a fire retardant in construction materials, electronic, plastic, textile, and other materials. Polybrominated biphenyls (PBBs) are used in consumer products such as computer monitors, televisions, textiles, and plastic foam [33]. PCBs are chemical materials that have both estrogenic and antiandrogenic effects. These compounds can disrupt thyroid hormone signaling and damage cell membrane receptors of steroid hormones. In addition, PCBs have sex-specific effects on hypothalamic mRNA expression, increasing lipid formation in lipid cells and serum E2 levels [3,34].

Dichlorodiphenyltrichloroethane (DDT) is a synthetic mixture of permanent pesticides formed from three isoforms. It has an estrogenic effect and disrupts the activity of thyroid-stimulating hormone (TSH) receptors [35,36].

Tributyltin (TBT) is a xenobiotic with many industrial applications, including broad spectrum biocides such as polyvinyl chloride catalyzers, agricultural fungicides, wood protection, and antifouling paints for marine vessels. TBTs activate ER and, by functioning as a strong activator of both retinoid X receptors and PPARγ, cause metabolic dysfunction and effects leading to obesity [37,38].

The effects of endocrine disruptors on the neuroendocrine system can lead to abnormal stress responses via mechanisms not yet fully understood. While there are some comprehensive studies on this topic, many chemicals have unknown effects and potential risks. Therefore, the effects of endocrine disruptors on human health must be carefully evaluated.

## 5. Neurobehavioral and neurodevelopmental effects

The recent increase in cognitive and behavioral disorders has led to concerns about the role of endocrine disruptors. Epidemiological data examining the potential link between endocrine disruptors and neurodevelopmental and neurobehavioral disorders has shown that exposure, especially in the prenatal and postnatal periods, can lead to severe neurodevelopmental problems in the fetus [39]. These problems include various developmental disorders such as autism spectrum disorder, attention deficit hyperactivity disorder (ADHD), mental development retardation, speech and language disorders, and developmental coordination disorder [39]. Adolescence is an important period when neuroendocrine activity increases, and exposure to chemicals that affect hormonal pathways at sensitive stages of neurodevelopment in this period could lead to adverse cognitive and behavioral outcomes [40].

Epidemiological data suggests that BPAs, phthalates, heavy metals, PFASs, PCBs, pesticides, organophosphates, and PBDEs have considerable effects on neurodevelopmental disorders leading to aggression, antisocial behavior, depression, somatization, and anxiety [40]. Although similar findings have not been found in different populations, prenatal exposure to BPA, bromide fire retardants, and organophosphate pesticides has been associated with an increase in externalizing behaviors, and BPA has been associated with inappropriate behaviors in children [41].

Exposure to BPA and phthalates can lead to lower academic performance in children, and intrauterine exposure to PCBs has been associated with lower intelligence quotient (IQ) [42]. In addition, prenatal phthalate exposure has been associated with ADHD development in children, poor speech and hand–eye coordination, and weak social relationships. Phthalates have antagonistic effects on thyroid hormone receptors and decrease in thyroid hormone levels [43]. In a population-based cohort study, prenatal exposure to a mixture of 26 different endocrine disruptors was related to a decrease in IQ. This effect was more evident in male children in particular, and it was emphasized that bisphenol F was the most important risk factor [44].

Animal and human studies have shown that exogenous disruptors can trigger the expression genes related to autism. However, in a systematic examination of 27 observational epidemiological studies with 77–1556 children aged 3–14 years, no correlation was found between prenatal exposure to endocrine disruptors and the subsequent development of autistic characteristics [45]. In those studies, exposure to PCBs, phthalates, phenols, parabens, organochloride and organophosphate pesticides, perfluoroalkyl substances, bromide fire retardants, and dioxins was examined. Although the authors found no relationship in that study, it could be misleading to conclude that there is no definite association between prenatal exposure and autistic characteristics. Attention was also drawn to the limitations of the studies with respect to representative exposure evaluations, small sample sizes, sex-specific effects, and insufficient evaluation of the endocrine disruptor mixtures [45].

Exposure to PCBs may increase the risk of ADHD. Although there are some studies supporting a relationship between ADHD and both BPA and PCBs, epidemiological research has provided conflicting results. One study of 7 birth cohorts in Europe found no relationship [46].

Despite the increasing evidence of an association between exposure to endocrine disruptor chemicals and adverse neurodevelopmental outcomes, epidemiological studies have not provided definitive results. This is made more difficult because of factors such as different population characteristics, the exposure duration, and exposure to a mixture of chemicals. Therefore, further experimental studies are of critical importance to be able to confirm these findings and explain the molecular mechanisms.

## 6. Conclusion

The relationship between endocrine disruptors and neurological diseases is supported by a growing body of evidence. Exposure, especially in the prenatal and early postnatal periods, plays a critical role in the development of neurodevelopmental and neurodegenerative diseases. Neurodevelopmental diseases such as ADHD and autism, and neurodegenerative diseases such as AD and PD are potential outcomes of long-term or low-dose exposure to endocrine disruptors. Previous studies have shown that common endocrine disruptors such as pesticides, heavy metals, and BPA can cause permanent damage to the nervous system. However, to fully understand the effects of these chemicals on human health, there is a need for high-quality, prospective studies. Understanding the molecular mechanisms of endocrine disruptors in the etiology of neurological diseases will be an important step toward the development of strategies to prevent these diseases.

## Footnotes

1 World Health Organization (WHO). Mental health: neurological disorders [online]. 2016. Available at: https://www.who.int/news-room/questions-and-answers/item/mental-health-neurological-disorders [accessed 27 February 2025]
